# Relation between digital tool practices in the language arts classroom and reading comprehension scores

**DOI:** 10.1007/s11145-022-10295-1

**Published:** 2022-05-07

**Authors:** Ladislao Salmerón, Cristina Vargas, Pablo Delgado, Naomi Baron

**Affiliations:** 1 ERI Lectura, University of Valencia, Av. Blasco Ibáñez, 21, 46010 Valencia, Spain; 2 Department of World Languages and Cultures, American University, Washington, USA

**Keywords:** Reading comprehension, Teachers’ practices, Digital devices, NAEP

## Abstract

Concerns about the negative effects of digitalization on students’ reading comprehension, empirically backed by recent meta-analyses, question the efficacy of digital tools in the language arts classroom. By analyzing data from 4 and 8th grade US students from NAEP 2017, we aimed to test the generalization of the negative association between screens use and reading comprehension test scores within language arts classrooms, and to identify teachers’ practices to support comprehension, which could reduce such a negative relationship. We used data from 149,400 4th grade and 144,900 8th grade students to predict their reading comprehension scores based on their frequency of use of digital devices in the language arts class, as well as on the specific learning activities performed with such devices. Results revealed that amount of daily use of digital devices was negatively related to scores on a reading comprehension test. In addition, teachers’ uses of digital tools to support students’ reading comprehension showed positive relations for student use of digital devices for reading projects, and negative relations for activities addressing specific reading skills, such as building and practicing vocabulary. We discuss these results in light of our current understanding of the effects of digitalization on reading.

## Introduction

Even before the COVID-19 pandemic, educational institutions across the world were moving rapidly to integrate digital reading tools in the classroom, under the assumption that computers and tablets will help students to improve both motivation and learning. Although strong concerns about the massive consumption of digital reading in education have been raised for more than a decade (Baron, 2015), not until the publication of recent meta-analyses has there been strong empirical evidence establishing challenges in the transition from paper to digital (Clinton, 2019; Delgado et al., 2018; Furenes et al., 2021; Kong et al., 2018). Those meta-analyses indicate the existence of a significant small effect size favoring comprehension in print (the so-called ‘screen inferiority effect’).

Explanations for the effect are still tentative, but preliminary evidence suggests that students tend to read on digital devices in a shallower way than with paper (i.e., the shallowing hypothesis: Annisette & Lafreniere, 2017). According to context models of text comprehension, during literacy tasks readers create an initial representation of the demands and affordances provided by the context, and use such representation to set reading goals and actions (Rouet et al., 2017). As young students tend to represent digital reading more as a leisure context than as a learning one, they may judge that digital reading requires low engagement and allows for distraction. Supporting this view, meta-analytical evidence indicates that students reading on digital devices tend to overestimate their comprehension to a higher extent than those reading on paper (Clinton, 2019). Such inadequate monitoring may lead to inefficient allocation of cognitive resources during reading. In a recent study, Delgado and Salmerón (2021) found that undergraduate students reading a long academic text in print tended to reduce their mind wandering thoughts when requested to do so under time pressure, as compared to when they were free to choose the amount of time on task. However, those reading the same material on a computer didn’t reduce their mind wandering in the time pressure condition.

Although evidence from the above meta-analyses suggests that paper-based reading has educational advantages over digitally-based reading when it comes to comprehension, there are theoretical and practical reasons to still recommend digital devices in the classrooms. First, from a theoretical perspective, we must keep in mind that previous studies reviewed in the above meta-analyses used single reading sessions to test differences in comprehension when using the two reading media. Since actual reading instruction may require extensive practice to be effective, a closer look at how digital tools are specifically used in reading classrooms is needed in order to test the generalizability of the screen inferiority effect described above to the instructional context. Second, from a practical perspective, it is evident that digital devices have become a necessary and powerful tool in schools. These devices can potentially support paper in building students’ comprehension. The goal of this study is twofold: to assess if a screen inferiority effect exists within the reading classroom pedagogical practices of a representative sample of lower and middle school students from the US, and to identify effective teachers’ practices to boost comprehension through digital devices within the classroom.

### Digital tools in classrooms and students’ reading comprehension

Prior large-scale studies have analyzed potential effects of reading medium within classrooms by focusing on the relationship between time spent using digital devices at school (measured by self-reported questionaires where students recorded their frequency of use of digital devices for different learning tasks at school) and students’ reading comprehension skills (measured by standardized tests containing series of texts and questions about them). Overall, the relationship tended to be small, with positive and negative associations across primary and secondary school years. Using data from 8th grade students from NAEP 1998, Wenglinsky (2005) found negative correlations between teachers’ use of computers in the classroom to teach reading (*r* = − 0.05) or to teach grammar/punctuation (*r* = − 0.05) and scores obtained by their pupils on a reading comprehension test. Using a different data set, Judge et al. (2006) analysed data from a representative sample of 8283 3rd grade students in the US, and found a negative correlation between teachers’ frequency of use of computers to teach reading and scores obtained by their pupils on a language test (which included a passage comprehension subtest; *r* = − 0.11) but a positive relation for frequency of computers use to access the Internet (*r* = 0.04). Evidence from large-scale studies are aimed at assessing trends, but they are not designed to provide specific explanations in terms of cognitive processing. Nevertheless, those patterns have been interpreted as evidence that teachers’ use of computers in the early 2000s was not effective in supporting students’ development of reading comprehension skills, though probably due to a lack of experience with computers.

Needless to say, the world of computer use in classrooms has changed dramatically over the years. More recently, data from 10th grade students who took the international PISA test for 2012 and 2018 revealed a negative relationship for using computers for practice and drill tasks at schools and reading comprehension scores (OECD, 2015, 2021). For example, the average difference in reading comprehension scores between students who reported practicing and drilling on a computer at school "never or hardly ever" versus “every day” was 63.5 (*SE* = 2.1; PISA 2012 average score was 496), after accounting for the socio-economic status of students and schools. In both the 2012 and 2018 samples, the relationship between frequency of browsing the Internet for schoolwork and reading comprehension scores was positive, with small but statistically significant relations. Thus, the more recent PISA evaluations suggest that, on the one hand, the generalized use of computers in classrooms is not positively associated with students’ reading comprehension development. On the other hand, some types of digital reading practices such as using the Internet for schoolwork positively relates to reading comprehension skills.

We should acknowledge that it is not possible to draw causal conclusions from correlational data, such as that coming from NAEPs and PISA datasets. In follow up studies using similar approaches, particular care should be taken to control potential confounding factors that could be involved in the negative as well as positive correlations between digital device use in schools and reading comprehension scores.

### Reading comprehension instruction with digital tools

Although most of the research just reviewed suggests a negative relationship between use of computers in the classroom and reading comprehension scores, the existence of null findings as well as positive patterns for specific purposes (Judge et al., 2006; OECD, 2015, 2021), offers the possibility that different teachers’ practices as well as instructional media to support reading comprehension might have different student outcomes when digital tools are used. Alternatively, positive relationships between computer use and reading performance could be due to other confounding effects, such as level of school resources (e.g., richer schools may have a lower ratio of students to computers with Internet connection). For example, the overall relation between reading digital texts at home and scores in the PISA 2018 reading comprehension test was positive, but became negative once schools’ and students’ SES was controlled for (OECD, 2021). In sum, further research is needed to identify the relationships between specific reading comprehension skills taught using digital tools in the classroom and reading comprehension achievement, as well as to control for potential confounding variables that may affect such relationships.

Which practices do instructors use to teach reading comprehension with digital tools in the classroom? Several studies have attempted to address this question. Based on a systematic review of research published between 2004 and 2015 in journals read by teaching practitioners, Yang et al. (2018) identified three major themes regarding use of technology for reading instruction: to increase students’ engagement and motivation, to present multi-modal information, and to use online collaborative reading activities. Recent evidence from PISA 2018 was used to investigate the extent to which the use of different school activities, not restricted to the language arts classroom, related to 10th grade reading comprehension (OECD, 2021). For the 10 school activities assessed, only the item “Browsing the Internet for schoolwork” showed a small positive relationship with reading comprehension. The rest of activities, including “Using school computers for group work and communication with other students” and “Practicing and drilling, such as for foreign language learning or mathematics”, showed medium to large negative relations. Looking at literacy instruction more generally, and not specifically for digital reading instruction, data from PISA 2018 identified teachers’ stimulation of students’ reading engagement as a key component of successful reading classes. The frequency with which a teacher “Poses questions that motivate students to participate actively” or “Encourages students to express their opinion about a text” was positively associated with reading comprehension (OECD, 2021).

The OECD, 2021 findings align with previous research documenting instructional practices of highly successful teachers (for a review, see Pressley & Allington, 2014). Successful reading teachers provide extensive opportunities for authentic reading tasks, in which students are given an open task that motivates their engagement, and then students must read texts to gather evidence, with the ultimate goal of providing a justified response to the task (Bransford et al., 2000). During such reading activities students could apply the comprehension strategies modelled by the teacher, to the extent that they may help students approach the task successfully. In other words, the purpose is to read in order to solve a problem. Comprehension strategies support this higher problem-solving goal. Such tasks are opposed to isolated drill and practice activities, where the purpose is to practice specific skills (e.g., find the main key words in a text). In these tasks, students apply a targeted comprehension skill, and reading the text becomes the context in which to reach the learning goal.

While authentic reading tasks appear relevant to students’ pedagogical success, we cannot assume they are representative of general practice. Indeed, studies regarding use of technology in classrooms suggest that most teachers don’t regularly use digital tools to support higher order reading activities (Fraillon et al., 2014; Orman & Padgett, 2017). For example, results from an international survey of representative samples of 8th grade teachers indicate that the two teaching practices most used with digital tools were presentation of information (33% of the teachers sampled) and using examples from the Internet to support a previous claim (21%; Fraillon et al., 2014). Less frequently, teachers had students use digital tools to work on short (20%) or long (14%) projects.

### The current study

The current study contributes to research on the relation between digitalized reading instruction and reading comprehension by analyzing data from NAEP 2017, a database containing representative samples of 4th and 8th grade students from the US. Using NAEP data allows us to identify the relations between both students’ reading time on digital devices and teachers’ instructional practices with digital tools to students’ reading comprehension skills, while having control over several potential covariates, ranging from students’ individual differences to teachers’ training. As the study is based on an existing database, it doesn’t allow for any experimental manipulation and thus could not be used to stablish causal claims. Nevertheless, we used an unprecedented set of covariates to minimize the possibility that the relationships observed were due to other uncontrolled factors.

We tested two hypotheses that correspond to the two main goals of the study. Hypothesis 1 is based on the expectation that the screen inferiority effect identified in experiments that used single reading sessions (e.g., Delgado et al., 2018) will also exist in the context of language arts classrooms, where reading is a regular activity throughout the school year. As an indicator of digital reading, we will look at frequence of student reading with digital tools relating to language arts activity (either at school or for homework). Specifically, hypothesis 1 states that frequency of time using digital tools for the language arts class will be negatively related to student reading comprehension achievement, both in the 4th and 8th grade.

Hypothesis 2 focused on the relationship between language arts teachers’ reading instructional practices with digital tools and student comprehension achievement. Based on previous studies on teaching excellence, hypothesis 2 predicts that instructional practices that emphasize authentic reading tasks when using such tools will be related positively to student comprehension achievement. Conversely, practices that emphasize drill and practice will be related negatively to reading comprehension. To control for alternative interpretations, such as the fact that classrooms that heavily use computers for drill and practice might be doing so intentionally because of needs of students in those classrooms, we also controlled for a set of student, teacher, and school variables (identified below) available in the NAEP databases that could have a significant relationship with reading comprehension achievement.

## Methods

### NAEP sample

This paper examines a portion of the 2017 NAEP database, a large-scale educational assessment that evaluates a number of academic subjects for primary and secondary school students in the United States. This iteration of the NAEP was administered between January and March of 2017 to 149,400 4th grade and 144,900 8th grade students. All test administration was done using tablet computers. In our research, we focus on the reading achievement of the students in each grade.

The 2017 NAEP included random samples of students selected to be representative of the US, including different states, large urban districts, and different regions of the country. In this probability multistage design, each school and student had a known probability of being selected. The school probabilities are proportional to the estimated number of students in the grade assessed. Each student only completed a subtest of NAEP items. Thus, a design called balanced incomplete block was used to assess the total framework across students. In addition, blocks appear an equal number of times in each of the block positions (either two or three).

### Variables

The study included student, teacher, and school variables as predictor variables, and reading achievement as a criterion variable. We also included several student and teacher variables as covariates.

#### Student predictor variable

*Digital device usage in the language arts class*. This index corresponds to student responses to the question “On a typical school day, how much time do you use a computer or other digital device to do your English/language arts schoolwork?” Note that the NAEP question asked about “English” as well as “language arts” schoolwork, since both terms are used in the US to describe literacy-intensive instruction. However, for simplicity, we use the term “language arts” from now on, since this is the more common term in US lower school education. Students were asked to use a 6-point Likert scale (0 = *Less than 30 min*; 1 = *About 30 min*; 2 = *About 1 h*; 3 = *About 2 h*; 4 = *About 3 h*; and 5 = *4 or more hours*). Similar items in previous versions of NAEP or in other educational assessments, such as PISA, focus on how frequently students engage in digital tasks at school overall (OECD, 2015). Compared to those other assessments, this NAEP item (introduced in 2017) is specific to the language arts classroom, where most instruction on reading and testing of reading comprehension take place. As such, the new question conforms to recent recommendations to increase the level of specificity of “screen time” assessments to improve its ecological validity (Kaye et al., 2020).

#### Teacher predictor variables

NAEP 2017 asked teachers of 4th and 8th grade students about use of digital tools for reading instruction. The question was formulated as follows: “In your [4th grade/8th grade] English/language arts class this year, how often do your students use a computer or other digital device to do each of the following?” A list of learning activities (see details below) was then given. For each activity, response options were: *Never or hardly ever* = 0; *Once or twice a year* = 1; *Once or twice a month* = 2; *Once or twice a week* = 3; *Every day or almost every day* = 4.

As the questionnaire included learning activities of different natures we decided to group them in two sets, according to their specific goal. The first set included activities where students’ goal was to practice specific reading components (vocabulary, fluency, comprehension). The second set included activities where students were provided an open question and asked to read to gather evidence and to provide a response (Guthrie et al., 2006; Polman, 2004).

*Student use of digital devices for reading components.* We created this variable by averaging the responses to different items on the use of digital tools for activities addressing specific reading skills. The NAEP question inquired about the following activities: *Build and practice vocabulary*, *Build reading fluency*, *Build reading comprehension*, *Practice spelling and grammar* (only available for the 4th grade sample), and *Access reading-related websites*.

*Student use of digital devices for reading projects*. This variable corresponded to teachers’ responses to the item *Conduct research for reading projects*.

#### Student covariates

*Gender* (0 = *Female*; 1 = *Male*).

*Disability status* (0 = *Not identified as student with disabilities*; 1 = *Identified as student with disabilities*).

*Eligibility for National School Lunch Program* (0 = *Not eligible*, 1 = *Reduced-price lunch*, 2 = *Free lunch*). This variable was used as an index of students’ family SES.

*Status as English Language Learner* (0 = ELL; 1 = not ELL).

*Reading self-efficacy*. This variable identified student belief regarding their capabilities in reading. It consisted of 6 items in 4th grade and 10 items in 8th grade. The students were asked to self-report how capable they felt they were in different aspects of reading (e.g., “*Explain the meaning of something you have read” and “Recognize the difference between fact and opinion in a text*”). These questions were answered using a Likert scale (0 = *I definitely can´t*; 1 = *I probably can´t*; 2 = *Maybe*; 3 = *I probably can*; 4 = *I definitely can*).

*Grit.* Grit can be defined as perseverance and passion for long-term goals. This variable consisted of 4 ítems (e.g., “*I keep working hard even when I feel like quitting*”) to be rated on a 5-point Likert scale (0 = *Not at all like me*; 1 = *A little bit like me*; 2 = *Somewhat like me*; 3 = *Quite a bit like me*; 4 = *Very much like me*”).

*In-class attention*. This variable measured student perceived ability to stay focused on what is relevant and resist distraction. The variable consisted of 4 items (e.g., “*I paid attention in class even when not interested*”), again using a 5-point Likert-type response format (0 = *Never or hardly ever*; 1 = *Less than half of the time*; 2 = *About half of the time*; 3 = *More than half of the time*; 4 = *All or almost all of the time*).

*Need for cognition*. Need for cognition can be defined as people’s tendency to engage in and enjoy difficult cognitive activity (Cacioppo & Petty, 1982). This 4-item measure (e.g., “I like complex problems more than easy problems”) asked students to indicate whether each statement was characteristic of them on a scale of 0 (*Not at all like me*) to 4 (*Very much like me*).

#### Teacher and school covariates

*Ratio of students to digital devices.* An index was computed as the ratio of students to combined number of laptops and tablets, using the following scale: 0 = *“* ≤ *1 student/device*”; 1 = “ > *1–2 students/device*”; 2 = *“* > *2–3 students/device*”; 3 = *“* > *3–5 students/device*”; 4 = *“* > *5 students/device*”. Accordingly, higher values indicated lower availability of devices.

*Teacher training in integration of digital devices into classroom instruction*. The teachers answered the question “*During the last two years, have you received training from any source in integration of computers and other technology into classroom instruction?*” Teachers’ responses were recoded as follows: 0 = *Have not*; 1 = *Yes or Already proficient.*

*Functionality of digital devices*. This variable comprises 3 questions addressed to teachers, using a 5-point Likert-scale, regarding how well desktop computers, laptop computers, and tablets worked in their school. The scale used was the following: 0 = *All computers/laptops/tablets are functional and operate quickly*; 1 = *All computers/laptops/tablets are functional, but some run more slowly than others*; 2 = *All computers/laptops/tablets are functional, but all ore almost all run slowly*; 3 = *Some of the computers/laptops/tablets do not operate and cannot be used*; 4 = *I don’t know*.

*Teaching limited by student behavior.* This variable consisted of 2 items addressing teaching limitations due to disruptive students and uninterested students, respectively, using a 3-point Likert-type response format (0 = *Not at all*; 1 = *Some*; 2 = *A lot*).

### Reading assessment as criterion variable

The NAEP assessment also measured reading comprehension. Students were asked to read passages and to answer questions about what they had read. The NAEP Reading Framework included two major text genres, literary and informational. Within the literary genre, the NAEP test includes fiction (e.g., adventure stories, contemporary realistic fiction, science fiction), literary nonfiction (e.g., personal essay, autobiographical sketches), and poetry (e.g., narrative poem, free verse, song). Within the informational text genre, the NAEP reading test includes expository (e.g., textbook, news article, encyclopedia entry), argumentative (e.g., letter to the editor, persuasive essay, editorial), and procedural texts (e.g., directions, timeline, schedule).

The number of texts and percentages of texts by genre differed between 4th grade (10 texts, 50% for each type of text) and 8th grade (13 texts, 45% literary and 55% informational). The passage length by grade was 200–800 words for 4th grade and 400–1000 words for 8th grade. Questions were presented in two formats: multiple-choice and constructed-response (both short and extended). In 8th grade, students spent about 60 percent of assessment time on constructed-response questions. For 4th grade, about 50 percent of the time involved constructed-response questions. Each student read two texts and answered approximately 20 questions about them. There were three types of questions. For locate and recall questions, students had to identify explicitly stated information or specific aspects of a story. For integrate and interpret questions, students had to infer relationships within and across multiple texts, by focusing on the main ideas, character motivation, or the theme of a story. Lastly, for critique and evaluate questions, students had to critically analyze the text by considering multiple perspectives or by evaluating the quality of the text (NAGB, 2017). Unfortuntally, the NAEP corpus available to us did not provide disaggregated data for each of the three question types, so we used the overall reading comprehension score.

The results used a 0–500 cross-grade scale, with *M* = 222, *SD* = 38 (4th grade) and *M* = 267, *SD* = 36 (8th grade; NAGB, 2017).

### Data analysis

Because each respondent was administered relatively few items in a scaling area in the NAEP, five plausible values (PVs) were computed on each subscale for each student. These plausible values were constructed from the results of a comprehensive marginal maximum likelihood (MML) regression equation. Then, to arrive at the dependent variable, it was necessary to consider all 5 PVs simultaneously with the aim of obtaining unbiased and stable estimates. Thus, we used the NAEP sampling weight in all MML regression models to test hypotheses. We included weights in the model to obtain unbiased estimates, after testing whether the weights were informative. For example, we found the level 1 and level 2 weights showed no equal means, and the variances were different from zero (Kim et al., 2014). In addition, the jackknife repeated replication method was used to estimate sampling variance of statistics in the NAEP data. The NAEP jackknife variance estimator (JVE) was based on 62 variance strata, resulting in a set of 62 replicate weights assigned to each school and student. This method provides approximately unbiased estimates of sampling variance (Shao & Tu, 1995). AM Statistical Software (American Institutes for Research, n.d.) was used for the statistical analyses, due to its capability of analyzing complex survey data containing plausible values.

In interpreting the results, readers should be aware that conceptually, the MML regression equation method we used is similar to a linear multiple regression model with the effects of covariates controlled for.

## Results

We first present descriptive evidence to provide a complete view of the dataset. Next, we present evidence from our modelling analyses to test the two hypotheses that guided our research: Our analyses aimed to test Hypothesis 1: frequency of time using digital tools for the language arts class will be negatively related to student reading comprehension achievement, both in the 4th and 8th grade and Hypothesis 2: instructional practices that emphasize authentic reading tasks when using such tools will be related positively to student comprehension achievement.

### Descriptive analyses

Descriptive statistics for the student and teacher variables are presented in Table 1. For students variables, we calculated (a) the percentage of students that responded to each response option, and (b) the average reading comprehension scores for those students that responded to a particular response option (e.g., how much time did they devote to using a digital device in the language arts class). For teachers variables, we calculated (a) the percentage of teachers that responded to each response option, and (b) the average reading comprehension scores for students in teachers classrooms, as a function of teachers’ response in each variable (e.g., use of digital devices for research for reading projects).

**Table 1:** Mean, standard deviation, and percentage of students for the composite reading achievement score for each school grade, for the student and teacher characteristics

Variable	Values	4th grade			8th grade		
		%	M	SD	%	M	SD
Gender	Male	51	219	39	51	262	36
	Female	49	225	37	49	272	35
Disability status	Yes	23	187	43	12	232	38
	No	87	227	34	88	271	33
SES^1^	Not eligible	43	236	33	47	277	33
	Reduced-price lunch	4	220	34	4	263	33
	Free lunch	47	207	37	42	252	35
ELL status	Yes	11	189	37	6	226	34
	No	89	226	36	94	269	34
Reading self-efficacy	Low	4	188	39	4	227	35
	Moderate	21	213	36	25	252	33
	High	49	238	31	63	277	31
Grit	Low	8	205	42	11	256	36
	Moderate	21	218	39	26	264	36
	High	60	229	33	56	271	34
In-class attention	Low	11	204	39	16	254	35
	Moderate	25	219	37	35	265	35
	High	52	232	33	42	274	34
Need for cognition	Low	18	216	38	29	258	34
	Moderate	26	222	37	32	266	35
	High	44	260	34	33	276	35
Digital device usage in the language arts class (time per day)	Less than 30 min	41	231	35	38	272	34
	About 30 min	34	226	35	29	266	36
	About 1 h	16	222	37	21	266	35
	About 2 h	5	216	39	7	267	36
	About 3 h	2	206	44	3	262	35
	4+ hours	3	192	42	3	251	37
Use of digital devices for research for reading projects	Never or hardly never	9	215	40	5	261	38
	Once or twice a year	28	223	36	26	267	35
	Once or twice a month	40	224	37	43	267	35
	Once or twice a week	18	224	38	19	269	35
	Every day or almost	6	223	40	6	269	36
Use of digital device for practicing reading components, item 1: Build and practice vocabulary	Never or hardly never	14	226	37	13	269	35
	Once or twice a year	6	226	37	9	270	35
	Once or twice a month	18	225	37	25	269	35
	Once or twice a week	37	222	38	35	266	35
	Everyday or almost	25	219	38	18	263	37
Use of digital device for practicing reading components, item 2: Build reading fluency	Never or hardly never	14	226	36	18	272	35
	Once or twice a year	6	228	37	11	270	34
	Once or twice a month	17	227	37	22	268	35
	Once or twice a week	34	222	37	28	265	35
	Everyday or almost	29	218	38	20	263	37
Use of digital device for practicing reading components, item 3: Build reading comprehension	Never or hardly never	8	226	37	12	272	35
	Once or twice a year	5	228	37	9	271	34
	Once or twice a month	15	227	37	20	268	34
	Once or twice a week	33	222	38	29	266	35
	Everyday or almost	40	220	38	30	265	36
Use of digital device for practicing reading components, item 4: Prectice spelling and grammar^2^	Never or hardly never	18	224	37	–	–	–
	Once or twice a year	7	227	37	–	–	–
	Once or twice a month	19	224	37	–	–	–
	Once or twice a week	35	222	38	–	–	–
	Everyday or almost	22	220	38	–	–	–
Use of digital device for practicing reading components, item 5: Access reading-related websites	Never or hardly never	11	224	37	13	269	35
	Once or twice a year	9	226	36	13	270	34
	Once or twice a month	25	225	37	13	268	35
	Once or twice a week	37	222	38	13	266	36
	Everyday or almost	19	218	38	13	264	37

^1^Percentages for these response options don’t sum 100 because because some options are not reported here (“Not available”; “Not participating”). ^2^Item used only in 4th grade

*Source*: U.S. Department of Education, National Center for Education Statistics, National Assessment of Educational Progress (NAEP) 2017 Reading Assessment

Reading comprehension scores were calculated with the total average scale composite. Overall, student characteristics were more related to reading comprehension scores than teacher or school variables. For example, the gap between students with the highest and lowest self-efficacy was 46 points (4th grade) and 47 points (8th grade) in the reading comprehension scores. By contrast, teachers with or without specific training to implement digital tools in the classroom resulted in only a 3-point (8th grade) or 4-point (4th grade) difference. Such patterns also emerged for the predictable variables. On the one hand, extremes in frequency of use of digital tools in the language arts classroom differed by 21 (8th grade) or 39 (4th grade) points, with higher reading comprehension scores for students who reported ‘lower or no use’. On the other hand, extremes in teacher use of digital devices for student research relating to reading projects only differed by 8 points (4th and 8th grade), with higher reading comprehension scores associated with teacher use for reading projects. Lastly, score patterns were mostly similar in 4th and 8th grade. One exception was the predictable variable frequency of use of digital tools in the language arts classroom, which showed a much higher relation with reading comprehension scores in 4th grade than in 8th grade.

### Model testing analyses 

The descriptive analyses suggested a strong negative relation between student frequency of classroom use of digital tools and reading comprehension, which provides initial support for Hypothesis 1. However, we can’t rule out the possibility that other relevant factors may partially explain this result. A modelling strategy is needed to isolate the relationships of our predictor variables. With this aim, we ran two weighted regression models with JVE for the 4th and 8th grade samples, with predictable variables and covariates as factors, and with students’ plausible values on the reading comprehension test as the dependent variable.^1^ Models estimates are presented in Table 2.

**Table 2:** Model estimates for the prediction of reading comprehension scores for 4th grade and 8th grade

Variables	4th grade		8th grade	
	Estimate (*SE*)	Z score	Estimate (*SE*)	Z score
Constant	206.22 (1.33)	155.31***	247.94 (2.34)	105.82***
Digital device usage in the language arts class	− 4.03 (0.18)	− 22.52***	− 2.07 (0.14)	− 14.65***
Gender	− 1.42 (0.37)	− 3.81***	− 4.89 (0.33)	− 14.90***
Disability status	− 28.10 (0.83)	− 34.02***	− 26.94 (0.61)	− 44.39***
SES	− 8.72 (0.21)	− 40.98***	− 8.16 (0.19)	− 42.54***
ELL status	− 22.89 (0.89)	− 25.62***	− 25.00 (1.08)	− 23.12***
Reading self-efficacy	11.10 (0.23)	47.65***	11.37 (0.25)	45.96***
Grit	0.83 (0.25)	3.32**	− 2.63 (0.20)	− 13.15***
In-class attention	1.79 (0.14)	12.69***	2.58 (0.23)	11.24***
Need for cognition	− 0.90 (0.17)	− 5.44***	2.19 (0.15)	14.84***
Ratio of students to digital devices	− 0.64 (0.19)	− 3.36**	− 0.54 (0.16)	− 3.30**
Teacher training in integration of digital devices	1.98 (0.58)	3.40**	2.28 (0.62)	3.67***
Functionality of digital devices	− 0.08 (0.24)	− 0.33	− 0.52 (0.24)	− 2.16*
Teaching limited by student behavior	− 4.63 (0.38)	− 12.09***	− 4.33 (0.36)	− 12.11***
Use of digital devices for project research	1.34 (0.27)	4.97***	0.90 (0.24)	3.77***
Use of digital devices for practicing reading components	− 1.03 (0.22)	− 4.74***	− 1.22 (0.19)	− 6.43***

4th grade model *R*^2^ = .41; *F*(15,106) = 620.382, *p* < .001. 8th grade model *R*^2^ = .37; F(15,110) = 716.12, *p* < .001. *p < .05; **p < .01; ***p < .001

*Source*: U.S. Department of Education, National Center for Education Statistics, National Assessment of Educational Progress (NAEP) 2017 Reading Assessment

The analyses resulted in significant models that accounted for a large portion of the variance, *R*^*2*^ = 0.41 (4th grade) and 0.37 (8th grade). The three predictable variables showed significant relations. To test Hypothesis 1 regarding students’ use of a digital device in the language arts class, we looked at the effects for students’ factors. Supporting Hypothesis 1 we found that, other things being equal, student frequency of classroom digital device use showed a negative relation in the 4th grade (estimate = − 4.03), which was also present, although half as large, in the 8th grade (estimate = − 2.07).^2^ Therefore, one additional point in student frequency of digital device usage in the language arts class is associated with 4.03 points lower on reading comprehension in 4th grade and 2.07 points in 8th grade. That is, the percentage difference between the average on reading comprehension (*M* = 222 in 4th grade and *M* = 267 in 8th grade) and a one point increase in student frequency of digital device usage in the language arts class is equivalent to stating a 1.8% and 0.8% decrease, respectively.

To test Hypothesis 2 regarding teachers’ reading practices with digital devices we looked at the effects for teachers’ factors. Supporting Hypothesis 2 we found that use of digital devices for research for reading projects showed a small positive relation with comprehension scores in both 4th grade (estimate = 1.34) and 8th grade (estimate = 0.90). Thus, a one-unit increase in the average use of digital devices for research for reading projects is associated with a gain of 1.34 (4th grade) and 0.90 (8th grade) points on reading comprehension scores. The percentage difference between both variables is equivalent to stating a 0.6% and 0.3% increase, respectively. Conversely, teachers’ use of digital devices for practicing specific reading components resulted in small negative relations for students in both 4th grade (estimate = − 1.03) and 8th grade (estimate = − 1.22). Hence, one additional point in this predictible variable is assosiated with 1.03 (percentage difference = 0.5%) and 1.22 (percentage difference = 0.5%) points lower on the NAEP scale in both grades. The relations for both types of practices were of similar magnitude, although of opposite direction.

Although they were not part of our hypotheses, we also explored the effects of the covariates, as they were included in the model to isolate its potential effect on students’ reading comprehension. Most of the covariates resulted in significant findings, with small to large relations in the expected direction. With regard to student status, we found that disability, SES, and classification as an ELL (English language learner) showed large negative relationships with reading comprehension both in 4th and 8th grade students, with lower achievement levels for students with a disability, lower SES level, or who were ELLs. Gender differences, in favor of girls, were approximately three times higher in 8th grade than in 4th grade. Among the student cognitive and emotional constructs analyzed, the positive relation of reading self-efficacy was by far the most relevant both in 4th and 8th grade, with estimates at least 5 times larger than those of other constructs such as grit, in-class attention or need for cognition. Teacher and classroom characteristics, teacher training, or knowledge about the use of digital tools in the classroom showed a small positive relation to student comprehension scores. by contrast, the extent to which teaching was disrupted by student behavior showed a larger negative relationship. Lastly, features relating to school characteristics—ratio of students to digital devices and the extent to which digital devices at the school function properly—showed negligible or non significant relationships.

## Discussion

This study contributes to our current understanding of the relations between use of digital tools and reading comprehension in representative samples of 4th and 8th grade US students in language arts classrooms. On the one hand, our analyses showed that, after controlling for a large set of student, teacher and school characteristics, even small daily amounts (here, 30 min) of use of digital devices in these classrooms are negatively related to scores on a reading comprehension test, and that this relation is almost double in 4th compared with 8th grade. On the other hand, teachers’ reading comprehension pedagogical practices with digital tools have both positive and negative relations with student reading achievement. While time devoted to reading projects with digital tools is positively related to student reading comprehension, time used to practice reading comprehension components such as vocabulary or fluency with digital tools is negatively related.

### Digitalization and student reading comprehension

The present study extends prior literature by examining specific use of digital tools in language arts classrooms. Consonant with earlier findings, our results indicate that the more that students used digital tools in their language arts class on a daily basis to perform more basic activities, the lower their reading comprehension achievement. Importantly, this association between reading comprehension achievement and the frequency of use of computers in reading instruction is large. It is similar to that of other well-known negative influcences that we also found in the NAEP dataset, such as the level of teaching limitations due to disruptive behaviors, or student self-reported in-class attention.

We suggest two complementary arguments that attempt to account for this finding. First, from the lenses of context models of text comprehension (Latini et al., 2019; Rouet et al., 2017), children’s habits developed through leisure activities using digital devices could be transferred to the way they read on such devices in school. A recent survey of US tweens and teenagers indicated that watching audiovisual mateial (53% of screen time), gaming (31%), and browsing websites (5%) are the activities that 8-to-12-year-old children do the most on screens (Rideout & Robb, 2019). Accordingly, children could represent the context of interacting with digital tools as a leisure activity, characterized by behaviors such as low effort and distraction. In learning contexts, using digital tools would activate such a leisure context model, which will favor non-optimal learning behaviors, including shallow attention and distraction.

Second, our results indicate that the negative association between the use of digital devices and student reading comprehension was twice as large in 4th grade as in 8th grade. Given that US teenagers use social media and web-browsing more often than younger children (Rideout & Robb, 2019), our unexpected result contradicts previous findings that support the shallowing hypothesis, showing that the more the readers use the digital devices for reading in a superficial way, the larger the negative association with depth of processing (Annisette & Lafreniere, 2017). Since our study was not designed to test developmental differences, we can at best speculate about the reasons behind our finding. We suggest two potential differentiating factors: pedagogical practices and student strategies. Regarding the first suggestion, our results show that teachers tend to use digital drill and practice tasks more often in 4th than in 8th grade, while the reverse holds true for reading projects. In addition, one would expect teachers to be presenting more sophisticated digital reading comprehension tasks to older students. All in all, the use of more surface-level reading activities with younger students could be partially responsible for the grade differences observed.

Regarding the second suggestion, 8th grade students are more cognitively mature than 4th graders, having a wider range of knowledge and an increased use of self-regulation strategies that are also more sophisticated (e.g., Zimmerman & Martinez-Pons, 1990). Accordingly, 8th grade students could have developed strategies to attenuate the shallow processing effect of on-screen reading (see also Salmerón et al., 2021).

It is also worth noting the possibility that the association found between frequency of digitized reading instruction, type of tasks, and student reading achievement could be somehow confounded with student prior reading skills. Instruction of specific reading components is used to train low-level basic skills and is especially fruitful for low achieving students (Lim et al., 2012). Thus, it is reasonable to consider that less skilled readers receive digitized drill and practice reading instruction more often than their skilled peers. Future research should look more closely at the pedagogical activities used by reading teachers, as well as student strategies to adapt to the characteristics of each reading medium (Goodwin et al., 2020). Such research should help in interpreting the developmental differences found in our study.

### Teachers’ practices to support comprehension with digital devices

Our results showed that the negative association between the use of digital devices and student reading comprehension is qualified by the type of learning activities for which the devices are utilized. We found that the overall negative association between reading comprehension and frequency of use of digital devices turned positive when looking at digital reading projects, as compared to drill and practice activities aiming to train lower-level reading skills. The fact that during projects students commonly engage in group discussions may promote their engagement in reading and deepen their understanding of texts (Polman, 2004; Yang et al., 2018). Furthermore, those activities could help students develop a context model of digital reading that includes sustained academic reading, as opposed to quick and leisure interactions. Constructing appropriate context models for digital reading would help students in setting appropriate goals and means to achieve them (Rouet et al., 2017). Future efforts should be directed to identify the psychological mechanisms, such as situational interest, group discussions or the creation of appropriate context models, that potentially help mediate the relation between digital reading projects and reading comprehension.

We note, however, that the NAEP 2017 dataset did not enable us to explore the interaction between the types of activities just described and digital vs. non-digital pedagogical implementations. Thus, we cannot rule out the possibility that the positive association between using digital devices for reading research projects and student reading comprehension is due to substantial practice with active engagement activities, regardless of the medium used. Further research, preferably using longitudinal designs, should address this issue by analyzing large-scale data including the frequency of practice of different types of reading activities with and without digital tools in order to measure potential interaction between instructional methods and medium. In this vein, recent results from the PISA 2018 assessment revealed that adolescents who favored reading print books scored higher on a text comprehension test than those who read more often in digital or in both media (OECD, 2021).

## Conclusions

Our study provides insights regarding use of digital devices as instructional tools for teaching reading in schools. Data from NAEP 2017 reveal that generalized use of digital technologies in education (here, in reading classrooms) appears susceptible to making the traditional mistake of adopting digital innovations without relying on evaluations of their effectiveness (Salmerón & Delgado, 2019). Our analysis suggests that many of the digitally-based activities performed in language arts classrooms in the US, particularly in lower grades, could well be hampering student reading development. Critically, future research will need to identify the reasons why teachers choose to use digital devices over other methods during their language arts classroom activities, and which factors influence those decisions.

## Footnotes

1 According to empirical evidence, parametric statistics may be acceptable for rating scale data such as that from Likert scales (Harpe, 2015; Norman, 2010).

2 In the manuscript we report unstandardized regression coefficients. The reason is that NAEP data must be analyzed using a special statistical software that considers complex sampling design. If these specific statistical packages are not used, results may be biased (for example, underestimated sampling errors). The National Center for Education Statistics recommends using AM Statistical Software to analyze NAEP data. Unfortunately, this program does not provide standardized regression coefficients (National Center for Education Statistics, n. d.).
